# Should we look for silent pulmonary embolism in patients with deep venous thrombosis?

**DOI:** 10.1186/1471-2261-14-178

**Published:** 2014-12-08

**Authors:** Maria José García-Fuster, Maria José Fabia, Elena Furió, Gernot Pichler, Josep Redon, Maria José Forner, Fernando Martínez

**Affiliations:** Internal Medicine, Hospital Clínico Universitario, Valencia, Spain; Research Institute, INCLIVA, University of Valencia, Valencia, Spain; CIBERObn, Carlos III Health Institute, Madrid, Spain; Servicio Medicina Interna, H.Clinico Universitario Valencia, Avda Blasco Ibañez n°17, Valencia, 46010 Spain

**Keywords:** Pulmonary embolism, Deep vein thrombosis, D-dimer

## Abstract

**Background:**

Asymptomatic or silent pulmonary embolism (S-PE) in patients with deep vein thrombosis has been the focus of numerous publications with the objective of determining the incidence of S-PE and assessing whether its existence has any clinical or therapeutic consequences that outweigh the risks associated with the diagnostic tests performed and the increased healthcare costs. The objectives were to assess the incidence of S-PE using computed tomography angiogram (CTA), to understand the epidemiological factors that might trigger embolism, and to assess whether D-dimer (DD) predicts the existence of S-PE’s.

**Methods:**

A prospective and consecutive assessment of 103 hospitalized patients with lower limb DVT in the absence of PE symptoms, using CT scan. DD was quantified before anticoagulation. The risk factors and characteristics of the DVT were studied. A three-year follow-up assessing risk recurrence and clinical outcome was performed.

**Results:**

The incidence of S-PE was 66%. In 77% of these cases, the main and lobar pulmonary arteries were affected. Iliac and femoral DVTs most often produced S-PE. ROC curve with a DD value higher than 578 ng/ml provided good sensitivity but low specificity to identify patients with S-PE. Diagnosis entailed higher hospitalization expenses. No significant recurrence rate of thrombotic events was observed in the S-PE group during the follow-up.

**Conclusions:**

The incidence of S-PE in lower-limb DVT is high, but in the absence of symptoms, diagnosis does not appear to be necessary, as there are no short- or long-term clinical or therapeutic consequences.

## Background

Asymptomatic or silent pulmonary embolism (S-PE) in patients with deep vein thrombosis was first described by Kistner [1]. Subsequently, it has been the focus of numerous publications with the objective of determining the incidence of S-PE and assessing whether its existence has any clinical or therapeutic consequences that outweigh the risks associated with the diagnostic tests performed and the increased healthcare costs [2–15].

To date the results have been very disparate. Findings show an incidence ranging between 11% and 59% [3–8]. All published studies have been retrospective, and most have used ventilation-perfusion (V/Q) scintigraphy as the method of diagnosis. Stein et al. [7], in a meta-analysis from 2010, which included 28 publications, found an average incidence of S-PE of 32%. Most published studies have found no advantage associated with diagnosis [10, 11] but the RIETE [8] group, however, recently observed that patients with deep venous thrombosis and S-PE had an increased recurrence of symptomatic PE during the first 15 days after thrombosis. This might suggest the need for stricter surveillance during this time period or perhaps the need for a vena cava filter.

The objectives of the present study were to prospectively estimate the incidence of S-PE in a group of patients with lower limb DVT, to assess the risk factors and characteristics of DVT that may have influenced their origin, and to assess the economic and clinical impact of diagnosis in both the short and long term.

## Methods

This was a prospective study of 103 patients, who were admitted consecutively to the Department of Internal Medicine, University Hospital Clinico of Valencia in 2009 for lower limb DVT without symptoms suggestive of pulmonary embolism (dyspnea, chest pain, hypotension, dizziness, fainting, sweating, tachycardia, low O_2_ saturation). The study was in compliance with the Helsinki Declaration and was approved by the Ethical Committee of the Hospital. Informed written consent was obtained from all participants.

The thrombosis were diagnosed by duplex Doppler ultrasound probe (Siemens Antares VF10-5), using the following criteria: visualization of a hypoechoic structure within the vein, passive distension of the vein or absence of local or distal compressibility and changes in venous flow dynamics [16, 17]. All patients underwent angio-pulmonary CT with a multidetector CT 16 and subsequent evaluation of the images in axial cuts and coronal reconstructions. The positive criterion was a filling defect, in place of the contrast, in a space in a lung artery. The extent of the contrast defect could be total or partial and, in the latter case, either as a ring or at an angle to the vessel wall [18]. Anthropometric data were collected: a) history of arterial risk factors (hypertension, diabetes, dyslipidemia) and b) venous risk factors (immobilization, surgery in the previous two months, hormone therapy). The morphological features of localization and extension of the thromboses and lung embolisms were assessed. The D-dimer was quantified before starting anticoagulation using D-dimer plus/BCS (quantitative turbidimetric assay) (Dade Behring Marburg GmbH). Normal values were considered to be <250 ng/ml.

All patients were treated according to protocol, which consisted of 3-5 days of low molecular weight heparin, adjusted for weight, followed by orally administered antagonists of vitamin K drugs (Acenocumarol) until an INR ≥2 was achieved, at that point, the heparin was removed. Patients received Acenocumarol for at least 6 months. The cost of care, along with the length of hospital stay, was calculated for patients with and without S-PE.

Patients were followed for three years and were assessed for hospital readmissions from causes of thromboembolic disease, other diseases and the presence of clinical data that might indicate the presence of pulmonary hypertension.

We calculated the means and standard deviations for absolute values. Continuous variables were compared with Student's t test, and categorical variables with chi-square or Fisher's exact test if necessary. The predictive power of DD as a suspected diagnosis using ROC curves and the area under the curve, and the Youden index was calculated.

## Results

### General characteristics of the study population

A total of 103 patients, 59 men and 44 women, were included. The age ranged between 17 and 90 years with a mean of 61.5 (SD 19). There were 46 hypertensives, 18 diabetics and 30 dyslipidemics. In 22 subjects, risk factors for venous risk found were trauma, surgery and hormone treatment. The thrombosis was ilio-femoral in 92 patients and distal, popliteal or more, in 11. It affected the right leg in 46 patients, the left in 57, and was bilateral in 5.

### Silent pulmonary embolism

From the 103 patients included in the study, S-PE was observed in 68, a prevalence of 66%. Age, sex, risk factors, and the venous thrombosis characteristics in triggering S-PE is reflected in Table 1, which shows that none of the analyzed factors had a greater presence in S-PE patients as compared to those without S-PE. The main pulmonary artery was affected in 35% of the cases, lobar arteries in 42% of the cases, and segmental ones in 23%.

**Table 1:** **General characteristics and deep venous thrombosis risk factors**

	All	S-PE	No S-PE	OR	p
Age (yr)	61.5 (19)	61.2 (19.4)	60.1 (18.8)	1.12 (-6.83-9.07)	0.78
Males	59 (57%)	42 (61.8%)	17 (48.6%)	0.59 (0.26-1.13)	0.22
***Risk factors***					
Venous^*^	22 (21.4%)	13 (19.1%)	9 (25.7%)	0.68 (0.26-1.80)	0.45
Hypertension	46 (44.7%)	30 (44.1%)	16 (45.7%)	0.94 (0.41-2.13)	1
Type 2 diabetes	18 (17.5%)	13 (19.1%)	5 (14.3%)	1.41 (0.46-4.36)	0.60
Dyslipidemia	30 (29.1%)	19 (27.2%)	11 (31.4%)	0.84 (0.35-2.06)	0.82
***DVT location***					
Ileo-femoral	92 (89.3%)	62 (91.2%)	30 (85.7%)	0.58 (0.17-2.56)	0.50
Poplitea	11 (10.7%)	6 (8.8%)	5 (14.3%)		
***DVT lateratity***					
Right leg	46 (44.7%)	28 (41.2%)	18 (51.4%)	1.5 (0.66-3.43)	0.40
Left leg	57 (55.3%)	40 (58.8%)	17 (48.6%)		
Bilateral	5 (4.9%)	5 (7.4%)	0	0.64 (0.55-0.74)	0.16

S-PE Silent Pulmonary Embolism; ^*^Venous risk factors (VRF) at least one of the following: immobilization, two months previous surgery, hormone therapy.

### Predictive value of D-dimer

**Figure 1:**
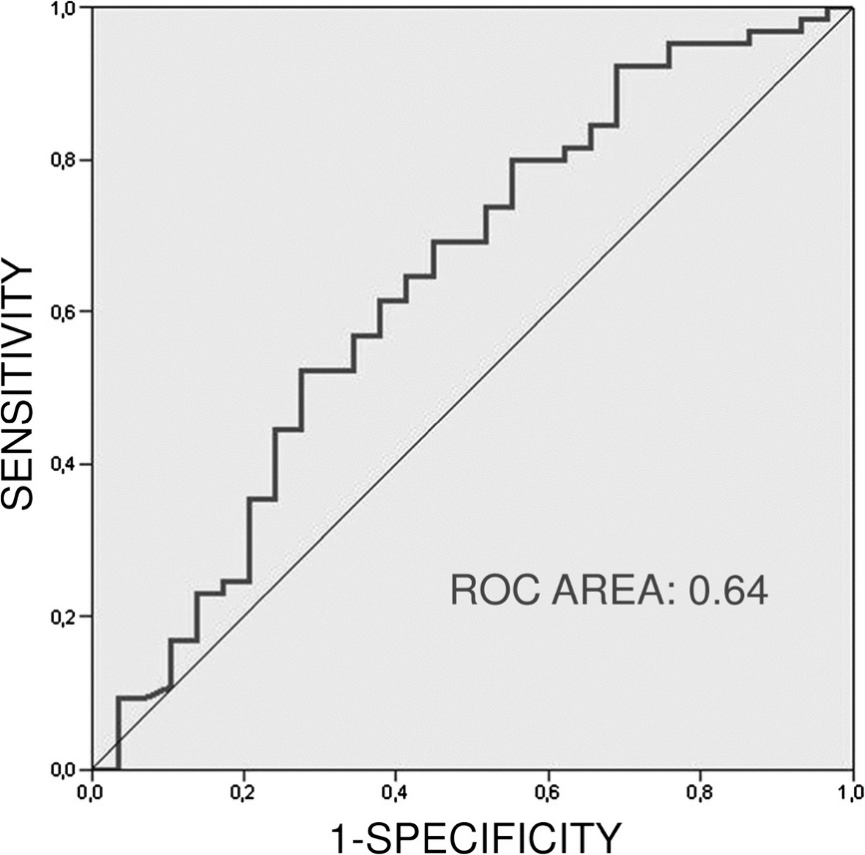
**Predictive value of D-dimer for pulmonary embolism.** ROC curve (see text).

The predictive value of the DD values was analyzed for the presence of S-PE in 94 subjects by ROC curves. Values >578 ng/ml were discriminant (area under the curve of 0.64 and p = 0.025) with a sensitivity of 80% and a specificity of 43% (Figure 1). The positive predictive value for this point was 76.5%, and the negative predictive value was 50%. Dividing the sample into three groups according to the DD values, ≤250 ng/ml, 251 to 578 ng/ml and >578 ng/ml, it was observed that the patients with S-PE were more frequently in the group with the higher DD value (p = 0.04) (Table 2).

**Table 2:** **Distribution of D-dimmer value in patients with and without silent pulmonary embolism**

	All	S-PE (%)	No S-PE (%)
DD <250 ng/ml	11	4 (6.3)	7 (23.3)
DD 251-578 ng/ml	14	9 (14.1)	5 (16.7)
DD >578 ng/ml	69	51(79.7)	18 (60)

DD (D-dimer), S-PE (silent pumonary embolism).

Chi-square p value 0.04.

### Follow-up

The average days of hospitalization were 9.86 **(SD 3.8)** for those without S-PE and 10.18 **(SD 4.7)** for those with S-PE. There were no clinical episodes suggestive of pulmonary embolism. During the three years of follow up, 25 patients were hospitalized for various reasons: 8 of the 35 who had no S-PE (23%) and 17 of the 68 who had S-PE (25%), with no significant differences. Of all readmissions, only 3 were for venous thromboembolism and 2 of these occurred in patients with a history of S-PE. The incidence of venous thromboembolic events was 0.96/100 patients/year for the S-PE group and 1.08/100 patient-years for the rest. All readmissions were for new DVT while none were for PE.

### Health care costs

All patients underwent a CT angiogram, which represented a single cost of 300€ more per patient. In cases in which S-PE was detected, the stay was prolonged by half a day, which entailed an additional cost of 275€.

## Discussion

In this study the prevalence of subjects with lower limb DVT which presented S-PE was 66%. The location of the thrombus in the iliac and femoral veins was frequent and the DD valuation had low specificity. The diagnosis of silent pulmonary embolism does not seem to offer any short- or long-term benefit and is an additional risk to the patient because of the administration and increased healthcare costs.

The percentage of silent pulmonary embolisms found in patients with DVT in this study is much higher than in previously published studies. Decousus et al. [3] evaluated 400 patients with DVT with an S-PE prevalence of 11%, most of whom had undergone a ventilation-perfusion scintigraphy. Monreal et al. [6] reported a 26% incidence while evaluating 1000 patients, also diagnosed by V/Q scintigraphy. Meignan et al. [5] evaluated 379 patients with the same method, and the incidence was 32%. This figure were similar to that found by Stein [7] in a meta-analysis in 2010, which noted that the diagnostic method used in most studies was V/Q scintigraphy, a method which has an 82% sensitivity for high or intermediate probability according the PIOPED (Prospective Investigation of Pulmonary Embolism Diagnosis) criteria. [12, 13]. Paul et al. [4] are the only authors found in the literature with an incidence of 59%, similar to that found in our study, because they used tomography or pulmonary angiography as their diagnostic methods.

In patients with S-PE, there appears to be a discrete higher prevalence in men than in women, a finding not corroborated with those from previous studies in which the percentage was the same for both sexes [6]. A higher incidence of S-PE was found in the elderly patients than in the younger ones, which is similar to that described by other authors [5, 14, 15]. This is not surprising given that thrombotic events increase with age. There was no evidence found that the cardiovascular risk factors, hypertension, diabetes mellitus and dyslipidemia, have any influence on the risk of S-PE. The same can be said for the transient venous thrombotic risk factors studied. Genetic thrombophilia was not included among the venous risk factors because a systematic study was not performed on all patients, only on the younger ones. The fact that the embolization of ilio-femoral thromboses is more frequent than the popliteal is to be expected, as it also is in symptomatic pulmonary embolisms [19–22].

Despite having no clinical impact, the main pulmonary and/or lobar arteries were affected in 77% of the cases. This is the authors’ main argument for defending the appropriateness of their diagnosis. It is known that the appearance of subsequent pulmonary vascular complications after embolisms, such as the development of pulmonary hypertension, impaired arterial-alveolar 0_2_ gradient, or the creation of dead spaces [2], depends largely on the affected vascular bed. The short and long-term monitoring conducted in this study, however, did not observe the appearance of any clinical data to suspect new PE or any other of the complications described. Nonetheless, it is possible that the increment in pulmonary arterial pressure may exist without any clinical repercussions during the time of observation.

D-dimer, a degradation byproduct of fibrin, has been used for years as a marker of thromboembolic events despite the handicap that results from its low specificity. Likewise, the different detection methods used to combine findings (ELISA or turbidimetric) hinder forming conclusions^23^. In principle, non-elevation is of great interest as an indicator to exclude the existence of pulmonary embolisms. The accepted significant figure of several authors is around 500 ng/ml, to consider their existence [13, 23–25]. The present study found that a value of 578 ng/ml was discriminative of the presence of PE but with a low sensitivity and even low specificity. 80% and 43%, respectively.

The hospital stay for patients with S-PE extends by an additional half day, suggesting that their existence does not complicate the illness. Hospital costs per patient, however, increase as a result of the cost of the CT angiography and the longer stay. While this this does not represent an important figure per patient, in itself, it can become one when considering the total number of patients who undergo CT angiography. The risk to the patient from the use of contrast and the unnecessary irradiation is an argument to consider, especially since it is usually performed in elderly patients whose renal function is frequently compromised [26, 27]. In these conditions, the potential risk could be avoided by using a combined perfusion scintigraphy with chest radiography as recommended by Sostman [28].

There are two arguments in defense of the investigation of a silent pulmonary embolism in patients with deep vein thrombosis. The first is that those who have suffered a pulmonary embolism are at increased risk of embolic recurrence, especially in the first 15 days [8]. The second is the concern that if PE is found during follow-up, it may be incorrectly diagnosed as a new PE resulting from a failure of anticoagulants. According to the ACCP guidelines [29], these would be situations which required the placement of a vena cava filter. That notwithstanding, the present study did not identify an increment in embolic events in the short or long term. In the three-year follow-up, the incidence of thromboembolic events was 2.8% for the same two groups of patients, lower than the previously reported by other authors for thrombosis [30]. What the present study did find was a greater number of readmissions for other diseases in the group of patients with S-PE, which could be interpreted as a higher comorbidity, since there were no differences in the risk of recurrence of thromboembolic disease.

The results of the present study should be considered within its strengths and limitations. A prospective design and 3 year follow-up give credit to the results. On the other hand the sample size is relatively small.

## Conclusion

This study found an incidence of silent pulmonary embolism of 66% for lower limb DVT, much higher than that reported previously. These are more frequent when the proximal veins were affected. The figure of DD has a low specificity in the diagnosis. According to this study the confirmation of S-PE appears unnecessary since its presence does not imply an increase in immediate or delayed morbidity or a change in treatment strategy.
